# Vaginal carriage of *Haemophilus influenzae* in a non-pregnant reproductive-age population

**DOI:** 10.1186/s12866-023-02885-y

**Published:** 2023-05-19

**Authors:** Meghana A Limaye, Sara Brubaker, Tara M Randis, Adam J Ratner

**Affiliations:** 1 Department of Obstetrics and Gynecology, New York University Grossman School of Medicine, New York, NY USA; 2 Department of Pediatrics, University of South Florida Morsani College of Medicine, Tampa, FL USA; 3 Department of Pediatrics, New York University Grossman School of Medicine, New York, NY USA; 4 Department of Microbiology, New York University Grossman School of Medicine, New York, NY USA

**Keywords:** *Haemophilus influenzae*, Perinatal pathogen, Vertical transmission, Neonatal infection, Vaginal microbiome

## Abstract

**Background:**

*Haemophilus influenzae* (*Hi*) is an emerging cause of early onset neonatal sepsis, but mechanisms of transmission are not well understood. We aimed to determine the prevalence of vaginal carriage of *Hi* in reproductive age women and to examine behavioral and demographic characteristics associated with its carriage.

**Methods:**

We performed a secondary analysis of stored vaginal lavage specimens from a prospective cohort study of nonpregnant reproductive-age women. After extraction of bacterial genomic DNA, samples were tested for the presence of the gene encoding *Haemophilus* protein d (*hpd*) by quantitative real-time polymerase chain reaction (PCR) using validated primers and probe. PCR for the V3-V4 region of the 16 S rRNA gene (positive control) assessed sample quality. Samples with cycle threshold (C_T_) value < 35 were defined as positive. Sanger sequencing confirmed the presence of *hpd*. Behavioral and demographic characteristics associated with vaginal carriage of *Hi* were examined.

**Results:**

415 samples were available. 315 (75.9%) had sufficient bacterial DNA and were included. 14 (4.4%) were positive for *hpd*. There were no demographic or behavioral differences between the women with *Hi* vaginal carriage and those without. There was no difference in history of bacterial vaginosis, vaginal microbiome community state type, or presence of Group B *Streptococcus* in women with and without vaginal carriage of *Hi*.

**Conclusion:**

*Hi* was present in vaginal lavage specimens of 4.4% of this cohort. *Hi* presence was unrelated to clinical or demographic characteristics, though the relatively small number of positive samples may have limited power to detect such differences.

## Background

Worldwide, sepsis and other infections cause approximately 15% of neonatal deaths [1]. Neonatal sepsis is divided into early-onset sepsis (EOS), defined as onset of symptoms before 7 days of life, and late-onset sepsis (LOS), which occurs between 7 days and 3 months of life. Many neonatal sepsis deaths occur on the first day of life, and preterm infants are at particularly high risk for sepsis and its sequelae.

EOS is frequently caused by vertical transmission of bacteria through infected amniotic fluid or from the mother’s vaginal canal during labor and delivery. LOS is generally thought to be the result of either vertical transmission or horizontal transmission from caregivers or the environment. In the United States, the most common pathogens for both EOS and LOS are Group B *Streptococcus* (GBS) and *Escherichia coli*, though with universal GBS screening and intrapartum antibiotic prophylaxis (IAP) the number of EOS cases caused by GBS has decreased [2, 3]. Concern remains that other pathogens resistant to antibiotics used in IAP may emerge as more frequent causes of neonatal sepsis.

*Haemophilus influenzae* (*Hi)* type b, a respiratory pathogen, was once a common cause of invasive bacterial disease in childhood, but with widespread vaccination, it has become a rare cause of invasive disease in the United States [4]. Both typeable and nontypeable strains of *Haemophilus influenzae* remain responsible for adult and neonatal pneumonia and can also cause severe female reproductive tract infection, when the organism’s presence in the vagina leads to upper genital tract infection through a break in anatomical barriers such as after surgery or delivery. In recent years there has been an increase in reported cases of neonatal sepsis due to *Haemophilus influenzae* [5–7]. The majority of these cases are EOS, suggesting vertical transmission as a potential source of infection. This hypothesis is additionally supported by another study reporting a significantly higher rate of invasive *Hi* infection in pregnant women [8].

The maternal vaginal microbiota represents a potential source of *Hi* in cases of neonatal infection, by vertical transmission during parturition. However, little is known about *Hi* in the vagina. It may be a transient vaginal colonizer, or it may be introduced from a respiratory or oropharyngeal source. Prevalence estimates range from 1.8/ 1000 in Scandinavian women in pregnancy^9^ to 7.3% in women with preterm premature rupture of membranes in Chile [10]. The rate of vaginal carriage of *E. coli*, which is known to be transmitted perinatally, is estimated at 13–32% [11–13] while GBS is estimated to be present in the vaginal microbiota of 18–40% of women [14]. Notably, the rate of *Hi* vaginal carriage in the U.S. in both pregnant and non-pregnant individuals has not been studied.

In order to further understand which women and neonates are at risk for sepsis caused by *Hi*, we evaluated the rate of vaginal carriage of *Hi* in a cohort of nonpregnant women.

## Methods

### Samples and parent study

We analyzed samples from a previously reported prospective study of 432 nonpregnant reproductive-age women. The Bacterial Vaginosis–Improved Diagnosis by ELISA and Sequencing (BV-IDEAS) study enrolled nonpregnant women aged 18–55 years seeking primary gynecologic care in New York City from July 2010-June 2012. After obtaining informed consent, 5 mL sterile saline vaginal lavage specimens were collected, refrigerated for transport (< 6 h), and stored at − 80 °C as previously described [15]. GBS status, determined by polymerase chain reaction (PCR) of vaginal lavage specimens, and vaginal microbiota community state subtypes have been reported for this cohort [15, 16]. Additionally, participants provided demographic information by self-report, including age, race, ethnicity, education level, income level, and behavioral characteristics including history of bacterial vaginosis, recent treatments with antibiotics or antifungals, and sexual practices. The original study was approved by the Institutional Review Boards at Columbia University Medical Center and Weill Cornell Medical College, and participants providing consent for future studies were included in the current analysis.

### DNA extraction and PCR

DNA extraction was performed on 500 µL of lavage specimen using the MagMax CORE Nucleic Acid Purification Kit on a Kingfisher Flex Purification System (ThermoFisher Scientific, Waltham, MA) after pretreatment with proteinase K, mutanolysin, and 1% lysozyme.

Samples were tested for the presence of the gene encoding *Haemophilus* protein d (*hpd*) by quantitative real-time polymerase chain reaction (PCR) using a validated primer/ probe set [17]. Each reaction mixture contained 1 µl of primer/probe mix with final concentrations of 500 nM primer and 250 nM probe, 10 µl of TaqMan Universal Master Mix II without UNG, 4 µl of RNAse-free PCR grade water, and 5 µl of extracted DNA. PCR conditions were as follows: 50.0^°^C for 2 min, 95.0^°^C for 10 min, followed by 95.0^°^C for 15s and 60.0^°^C for 1 min for 50 cycles. PCR was performed in 96-well plates on an Applied Biosystems StepOne Real-Time PCR System. Fluorescence threshold was set at 0.2 and C_T_ values less than 35 were considered positive. A positive control of *Hi* genomic DNA extracted using the MoBio Powersoil DNA extraction kit (Qiagen) was included on each run. Negative controls with master mix, primer, probe and no template were also included.

To assess sample quality, PCR for the V4-V5 region of the 16 S rRNA gene was performed using specific primers and probe as described [18]. The same conditions were used for PCR as described above. PCR for the 16 S rRNA gene and the *hpd* gene was performed on the same run for each sample. Samples were determined to have sufficient bacterial content if the C_T_ was < 35 for the 16 S rRNA PCR.

### Sequencing

PCR products from samples that had sufficient bacterial content and were positive for *hpd* were run on a 2% agarose gel. Bands corresponding to *hpd* were cut from the gel and DNA was extracted using a QIAquick gel extraction kit (Qiagen, Valencia, CA). Sanger sequencing of the PCR products of positive samples was performed (Genewiz, South Plainfield, NJ) to confirm the presence of *hpd*.

### Statistical analysis

Demographic and behavioral characteristics of women with and without vaginal carriage of *Hi* were compared. Missing data are due to participant non-response to the survey questions in the parent study. Vaginal microbiome community state types and presence of GBS was also compared between groups. Chi-square, Fisher’s exact test, and t-test were used with p < 0.05 set as the level of statistical significance. All statistical analysis was performed using SPSS Version 24 (IBM Corp, Armonk, NY).

## Results

**Fig. 1:**
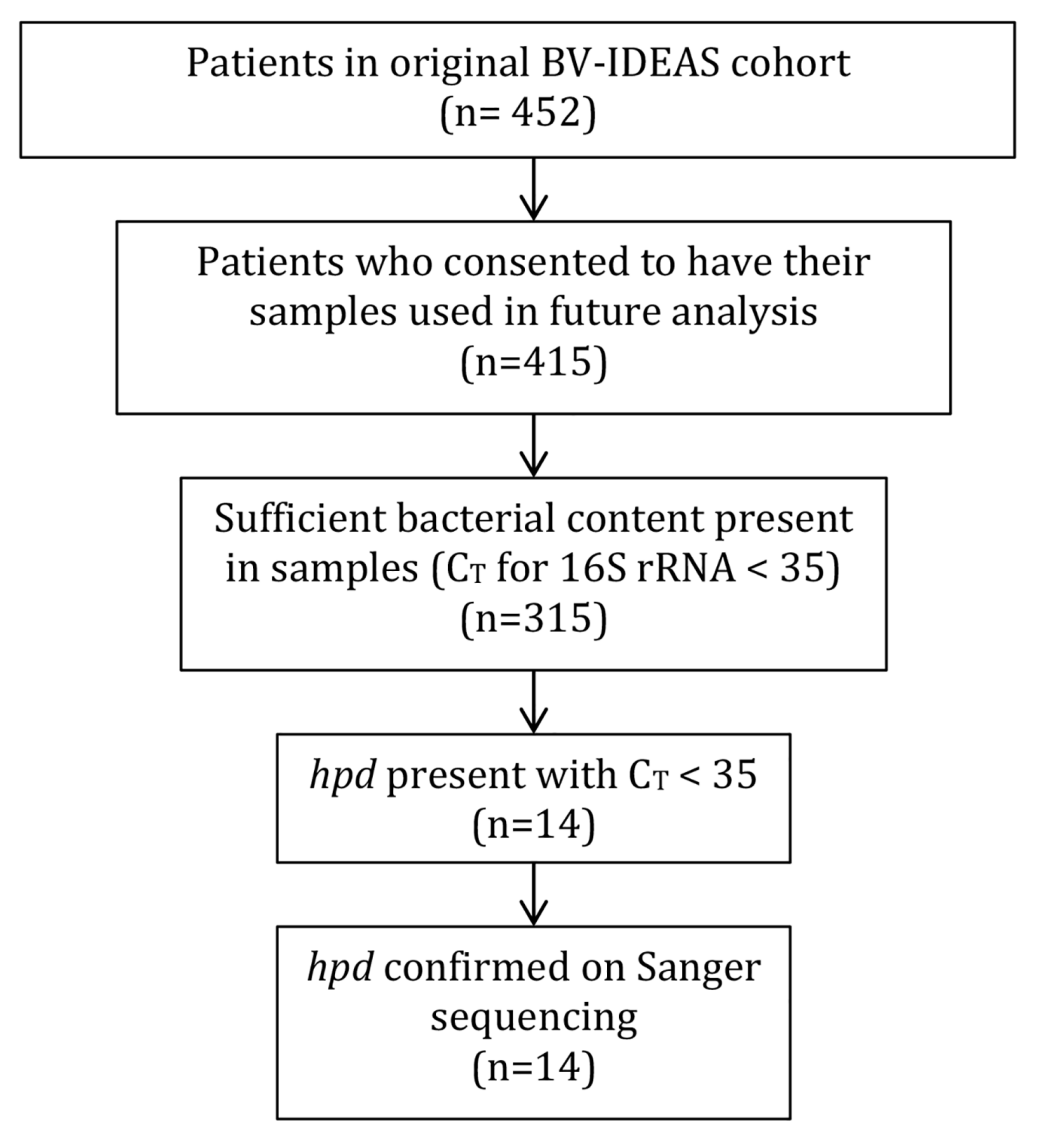
Study flow diagram

452 subjects were included in the original cohort. Of those, 415 had provided consent for future analysis and had samples were available for this study. 315 (75.9%) had sufficient bacterial DNA present, as determined by real time PCR with C_T_ < 35 for the 16 S rRNA gene and were included in the analysis. 14 samples (4.4%) were positive for *hpd* by real-time PCR with C_T_ < 35, which was confirmed by Sanger sequencing of the PCR products. Study flow diagram is shown in Fig. 1.

There were no demographic differences between the women with *Hi* vaginal carriage and those without (Table 1). There was no difference in presence of GBS, history of bacterial vaginosis, or vaginal microbiome community state type in women with and without vaginal carriage of *Hi* (Table 2). Additionally there were no statistically significant differences in sexual behaviors such as receptive oral sex noted (Table 2). Characteristics of participants’ partners are reported in Table 3. No difference was seen in partner characteristics in the two groups.

**Table 1:** Demographic characteristics

	*hpd* negative (n = 301)	*hpd* positive (n = 14)	p-value
Age	32.8 (10.1)	35.4 (6.1)	0.33 ^a^
Race/ Ethnicity White Black Hispanic/ Latino Asian American Indian/ Alaska Native Pacific Islander/ native Hawaiian Middle Eastern	48 (15.9) 105 (34.9) 127 (42.2) 16 (5.3) 0 (0) 2 (0.7) 3 (1)	3 (21.4) 6 (42.9) 5 (35.7) 0 (0) 0 (0) 0 (0) 0 (0)	0.90
Education Less than high school Some high school High school grad GED	181 (60.1) 94 (31.2) 9 (3) 17 (5.6)	6 (42.9) 7 (50) 0 0	0.46
Insurance None Medicaid Family health plus Private Other	11 (3.7) 144 (47.8) 21 (7.0) 2 (0.7) 123 (40.9)	0 (0) 10 (71.4) 3 (21.4) 0 1 (7.1)	0.05
BMI	*(n = 296)* 27.7 (7.3)	*(n = 14)* 30.1 (6.5)	0.25 ^a^

Data presented as n (%) and chi-square or Fisher’s exact test p-value, unless otherwise indicated

^a^ p-value from two-sided t-test

**Table 2:** Clinical and behavioral characteristics

	*hpd* negative (n = 301)	*hpd* positive (n = 14)	p-value
Positive GBS	62 (20.6)	1 (7.1)	0.32
Antibiotic use in past month	33 (11.0)	1 (7.1)	0.65
Douching day of exam	21 (7.1)	3 (21.4)	0.09
Ever pregnant	247 (82.1)	13 (92.9)	0.48
Had sex in past month	(n = 294) 188 (63.9)	(n = 14) 9 (64.3)	0.98
Had receptive oral sex in past month	(n = 289) 125 (43.3)	(n = 13) 7 (53.8)	0.45
Number of oral sex partners in last month ^b^	(n = 295) 0 (1.0)	(n = 14) 0.5 (1.0)	0.45^c^
Lifetime history of yeast infection	200 (68.3)	10 (83.3)	0.27
Lifetime history of bacterial vaginosis (BV) (n = 314)	68 (23.9)	5 (38.5)	0.48
Ever received treatment for BV	(n = 68) 66 (97.1)	(n = 4) 4 (100)	0.94
Sexual identity Heterosexual Lesbian Bisexual Other	(n = 295) 270 (91.5) 5 (1.7) 19 (6.4) 1 (0.3)	(n = 14) 14 (100) 0 0 0	0.73
Vaginal microbiome community state type I II III-A III-B IV-A IV-B V	41 (13.6) 11 (3.7) 12 (4.0) 61 (20.3) 11 (3.7) 153 (50.8) 11 (3.7)	2 (14.3) 0 0 5 (35.7) 1 (7.1) 6 (42.9) 0	0.81

Data presented as n (%) and chi-square or Fisher’s exact test p-value, unless otherwise indicated

^b^ Data presented as median (IQR)

^c^ p-value from Mann-Whitney test

**Table 3:** Partner characteristics

	*hpd* negative	*hpd* positive	p-value
Male partner	(n = 300) 294 (98)	(n = 14) 14 (100)	1.00
Partner circumcised	(n = 278) 100 (36)	(n = 13) 6 (46.2)	0.56
Partner race/ ethnicity White Black Hispanic/ Latino Asian American Indian/ Alaska Native Pacific Islander/ native Hawaiian Middle Eastern	(n = 296) 54 (18.2) 123 (41.6) 104 (35.1) 9 (3.0) 0 1 (0.3) 5 (1.7)	(n = 14) 1 (7.1) 7 (50) 6 (42.9) 0 0 0 0	0.86
Partner with STD in the past year	(n = 253) 14 (5.5%)	(n = 13) 1 (7.7%)	0.54
Relationship with partner Husband Boyfriend Father of children Wife/ female life partner Girlfriend	(n = 291) 224 (77) 14 (4.8) 7 (2.4) 38 (13.1) 8 (2.7)	(n = 12) 11 (91.7) 0 1 (8.3) 0 0	0.36

Data presented as n (%) and chi-square or Fisher’s exact test p-value

## Discussion

*Haemophilus influenzae* DNA was present in vaginal lavage specimens of 4.4% of nonpregnant women. The presence of *Hi* was unrelated to clinical or demographic characteristics in this cohort, though the relatively small number of positive samples may have limited power to detect such differences.

The rate of *Hi* vaginal carriage in this study is similar to the rates seen in the literature in pregnant and non-pregnant women. In pregnancy, the rate of vaginal carriage of *Hi* in Denmark in 1989 was 1.8/ 1000 women presenting in labor, [9] while in a study from Chile in 1992–1998, *Hi* was isolated from the vaginal culture specimens of 7.3% of 110 women with PPROM [10]. In both studies the presence of *Hi* was determined by culture and confirmed by PCR of the 16 S rRNA gene. A study of 510 pregnant women in Italy found the overall prevalence of carriage of the *Haemophilus* genus to be 9% using culture-based methods confirmed by sequencing the full-length 16 S rRNA gene. However, only *H. parainfluenzae, H. pittmaniae and H. haemolyticus* were present with no *Hi* detected [19].

Outside of pregnancy, the rate of vaginal carriage of *Hi* appears to be similar. A study of 216 nonpregnant women in Australia using a multiplex PCR assay for 14 microbial species and the *Hi gyrR* gene found that *Hi* was present in 5.1% of the vaginal swab samples [20, 21]. An individual participant meta-analysis of the vaginal microbiome data of 1,163 women noted the presence of *Hi* at low levels, though the prevalence of *Hi* from the 16 S rRNA microbiome sequencing data was not reported [22]. In our previously reported sequencing analysis, *Hi* was not present at detectable levels in the current samples [15]. We speculate that levels of *hpd* DNA were likely too low to be detected by the sequencing pipeline that was used in that report. Targeted PCR based amplification as performed in this study allowed identification of *hpd* at lower levels.

This study has several strengths. It is a large cohort with detailed information about individual clinical and behavioral characteristics, and validated primers and probe from the CDC were used for evaluation of the presence of *Hi.* The *hpd* protein that was targeted is specific to *Hi* and more accurately identifies *Hi* than the 16 S rRNA or the culture-based techniques used in most prior studies. Sanger sequencing was performed on samples that were positive for *hpd* to confirm its presence, decreasing the risk that contamination or background signal led to false positives. The use of PCR enhances the ability to detect low numbers of *Hi* as compared to vaginal microbiome studies utilizing 16 S rRNA sequencing. While this ability to detect low levels of *Hi* is a strength of our study, these levels may in fact be too low to cause clinically significant infection. Therefore, we are unable to draw any conclusions about the infectivity of this bacterial species based on this data, and additional research is needed to further elucidate the ability of *Hi* to cause reproductive tract and neonatal infection.

There are also several limitations to the study. The number of samples positive for *Hi* is low, limiting our ability to make meaningful conclusions regarding demographic and clinical risk factors for the vaginal carriage of *Hi*. The samples may have degraded during storage, causing some to have a low bacterial content. We included only samples with sufficient bacterial content (16 S rRNA C_T_ < 35) but there may have been *Hi* present in samples that were excluded. It is unlikely that samples with or without *Hi* present would degrade at different rates, thus sample degradation is unlikely to have biased our conclusions. There may also be some selection bias present as we only analyzed the samples of women who consented to future research. Though unlikely, there may have been a different prevalence of *Hi* in women who declined future study participation. The samples used in this analysis were collected by vaginal lavage, which may not correlate exactly with vaginal swabs as were used in other studies. However, since the prevalence of *Hi* in our population is similar to that seen in other studies, this method of collection of vaginal samples does not appear to limit the applicability of our study.Finally, it is possible that some samples in this study that were positive for *hpd* may in fact contain *H. haemolyticus*. The ability to differentiate the two species by culture-based or molecular techniques is limited and rare cases of invasive infection due to *H. haemolyticus* have been reported. Though *H. haemolyticus* is typically negative for the *hpd* gene, sequencing has shown that it may be present in some strains, making *H. haemolyticus* very difficult to distinguish from *Hi* [23–25].

## Conclusion

*Haemophilus influenzae* was present in vaginal lavage specimens of 4.4% of nonpregnant women. The presence of *Hi* was unrelated to clinical or demographic characteristics in this cohort, though the relatively small number of positive samples may have limited power to detect such differences. Further studies should assess for the presence of *Hi* as a colonizing organism in pregnant women and well neonates. Persistence of vaginal carriage throughout pregnancy, transmissibility of *Hi* to the neonate and the percentage of exposed neonates who become clinically ill all remain unknown and should be assessed.

## Data Availability

The dataset used during the current study is available from the corresponding author on reasonable request.
